# Ex vivo fluorescence confocal microscopy: chances and changes in the analysis of breast tissue

**DOI:** 10.1186/s13000-022-01240-5

**Published:** 2022-06-28

**Authors:** Maja Carina Nackenhorst, Mohammad Kasiri, Bernd Gollackner, Heinz Regele

**Affiliations:** 1 Department of Pathology, Medical University of Vienna, Vienna, Austria; 2 Department of Surgery, Medical University of Vienna, Vienna, Austria

**Keywords:** Digital Pathology, Ex vivo fluorescence confocal microscopy, Gynecopathology, Frozen Sections

## Abstract

**Background:**

Rapid histologic diagnosis of frozen sections is essential for a variety of surgical procedures. Frozen sections however, require specialized lab equipment, are prone to freezing artifacts and are not applicable to all types of tissue. Adipose tissue is especially difficult to process in frozen sections. Although these limitations are well known, no alternative method for microscopic tissue analysis that might replace frozen sections could be established. Our objective was to evaluate whether tissue imaging based on ex vivo fluorescent confocal microscopy (FCM) is applicable for rapid microscopic assessment of breast tumors specimens with abundant adipose tissue.

**Methods:**

We evaluated 17 tissue samples from mastectomy specimens, rich in adipose tissue, submitted to the department of pathology at the Medical University of Vienna. We conducted our study on the FCM VivaScope® 2500M-G4 (Mavig GmbH, Munich, Germany; Caliber I.D.; Rochester NY, USA).

**Results:**

When comparing FCM to frozen sections, we found a very similar overall processing time for FCM images and frozen sections respectively. Image quality was mostly superior to frozen sections (especially for adipose tissue and nuclear detail) but inferior to H&E stained FFPE sections. Limitations of the technology were uneven coloring, invisibility of ink applied for marking tissue margins and distortion artifacts if too much pressure is applied to the tissue.

**Conclusion:**

FCM has the potential to expand the application and usefulness of rapid tissue analysis as speed is comparable and quality exceeds that of frozen sections especially in tissues rich in adipose cells such as breast specimen.

## Introduction

Frozen sections are the gold standard for rapid histologic assessment of tissue. They are widely used for intraoperative histologic diagnostics especially in oncologic surgery. Unfortunately, frozen sections are prone to artifacts, cannot be applied to adipose tissue due to its propensity to tear and crinkle, and their overall quality is generally far inferior to sections from paraffin embedded tissue [1]. Additionally, frozen section diagnostics is a costly procedure since it requires specialized lab equipment (cryostat, staining solutions, appropriate work benches, etc.) and a skilled technician. It is thus restricted to centers with adequately equipped pathology laboratories. The tissue is also lost for further investigations. Despite these limitations, no equivalent method of investigation could be established in clinical practice so far.

Direct microscopic assessment of tissue without sectioning is technically feasible. The application of technologies like Raman spectroscopy, light-sheet microscopy or fluorescent confocal microscopy (FCM) to human tissue specimens is under development and yielded promising results in some areas [2, 3]. Very few systems however are commercially available and might be suitable for medical diagnostic applications. FCM is of particular interest as it has already been successfully employed in clinical dermatology and urology for investigating skin and prostate biopsies with regards to rapid diagnosing the absence or presence of malignant tumors and other pathologies such as cutaneous vasculitis [4–8]. Another study showed high agreement of FCM analysis of biopsies from a variety of tissues with H&E staining [9].

FCM allows rapid virtual microscopic analysis of tissue, directly yields digital images ready for telepathology applications and is also suitable for specimens rich in adipose tissue like breast tissue and pancreatic tissue that is very difficult to satisfyingly depict in frozen sections.

Adipose tissue has always been a challenging tissue to display in frozen sections due to its propensity to tear and fold over. As our center receives frequent breast specimen for analysis, we chose breast tissue for our study comparing FCM to standard frozen sections to evaluate whether the procedure can be performed in a satisfactorily fashion and in a time frame comparable to frozen sections. A study by Elfgen et al. showed promising results for comparing breast specimen biopsies to H&E images utilising a different FCM platform [10].

## Material and methods

### Patients

This study was approved by the local Ethical Committee (protocol number 1872/2019). Patient data was pseudonymized for this study and the code was separately stored and was only accessible by authorized personnel. We evaluated 13 tissue samples from breast specimen send to the department of pathology obtained from ablation procedures. We performed biopsies on 4 samples with a 16 gauge needle to mimic biopsy conditions.

### Specimens and staining procedure

**Fig. 1:**
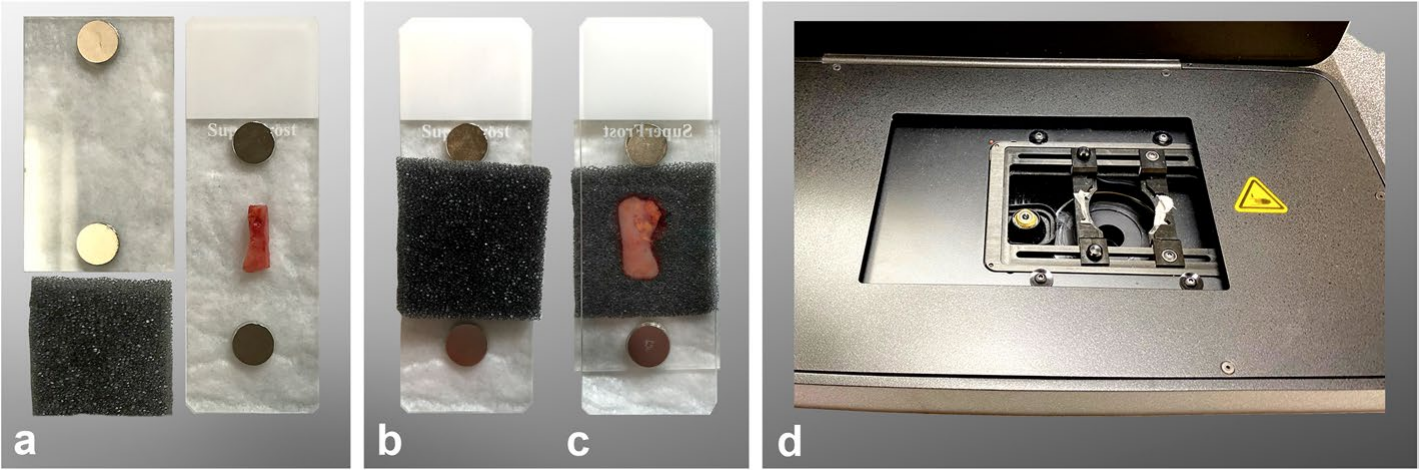
Sample preparation for scanning, **A** Glass slide with tissue sample and sponge, **B** and **C** magnetic coverglass applies pressure to tissue sample on sponge, **D** Stage of the Vivascope 2500M-G4

The native tissue was processed immediately after acquiring the samples and at most no later than 15 min after harvesting. Samples were cut to a size comparable to tissue samples for frozen sections. Staining of tissue for highlighting nuclei was performed according to a protocol provided by the manufacturer of the instrument: Each sample was first immersed in PBS buffer (1:10 dilution, pH 7.2, Morphisto, Frankfurt am Main, Germany) for 30 s, followed by 96% alcohol (VWR chemicals, Fontenay-sous-Bois, France) for 30 s, then stained with 1% solution of acridine orange dye (Morphisto, Frankfurt am Main, Germany) for 30 s and finally washed in PBS buffer solution for 30 s. All tissue samples were then placed on a glass slide and covered with sponges that apply gentle pressure to the tissue in order to ensure complete contact between tissue and glass slide. The sponge is held in place by a magnetic-mounted coverglass (Fig. 1). Each slide was positioned in the FCM stage for image acquisition. After the acquisition, the samples were put in biocassettes and immediately fixed with in buffered formalin (7.5%) for conventional histopathological workup and evaluation.

### Confocal microscope

We conducted our study on the FCM VivaScope® 2500 M-G4 (Mavig GmbH, Munich, Germany; Caliber I.D.; Rochester NY, USA) [11]. This confocal microscope has two lasers with different wavelengths: one with 488 nm (fluorescence) and one with 785 nm (reflectance). Both lasers were applied to each sample for visualizing AO stained nuclei (488 nm) and tissue structure (785 nm). The FCM has a maximum examination depth of 200 μm, a vertical resolution of at least 4 μm, a magnification of × 550 and a maximum scan size of 25 × 25 mm [11].

The final image delivered by the instrument is a whole specimen scan based on stitching of mosaic images (square-shaped images of 1024 × 1024 pixels). The laser filter is equipped with a 38x, 0.85 numerical aperture water immersion objective lens. To analyse the images VivaScan® (Version 11.0.1140 Mavig GmbH; Caliber I.D), VivaBlock®, and VivaStack® are necessary software tools for generating whole tissue scans, the acquisition of multiple images in the X/Y directions within a single focal plane, and a survey of multiple focal planes along the Z axis, visualizing tissue at various depths, respectively. The grayscale fluorescence and reflectance mosaics are digitally stained similar to the aspect of H&E staining in order to make the resulting image as easy to interpret for the pathologist as possible. Individual images are then assembled to a whole specimen scan via a built-in algorithm.

### Image evaluation

All images were pseudonymized and stored on a separate system. The images were separately analyzed by two pathologists.

Analysis was performed based on the following parameters:

1. Structural integrity: The digital images were evaluated for tears or other forms of structural defects like compression.
2. Staining properties: Staining properties of digital specimens were compared to standard H&E staining regarding distribution, colour and intensity.
3. Morphological detail: Tissue architecture, nuclear morphology and cytoplasmic detail as well as overall cell structure were evaluated and compared to frozen sections and/or paraffin section of the same specimen.
4. Final diagnosis: presence and type of tumor.

### Histological evaluation

Samples were embedded in FFPE and 3 μm sections were cut by experienced technicians. Sections were then stained with hematoxylin–eosin according to standard procedures. Standard diagnostic criteria for detection of neoplastic tissue and assessment of malignancy were applied.

## Results

### Processing time

Time needed for scanning varies on tissue sample size. On average, a scan was available after 4–6 min for the pathologist to evaluate (2 min for the staining procedure and typically 2–4 min for scanning the specimen). Scanning time of course increases with the size of the sample. The samples analyzed in this study mostly did not exceed a size of 10 mm x 10 mm.

### Structural integrity

The overall impression of tissue texture was comparable to conventional frozen section histology, however representation of tissue structures was focally discontinuous in all 17 cases. The incompletely represented areas were small and located both within the tissue and at tissue borders and generated the impression of uneven staining that was more pronounced in some areas and weaker in others. In most instances, discontinuous representation likely resulted from lack of mechanical pressure leading to areas of tissue being detached from the glass slide and thus not represented in the digital image. Correct diagnosis of malignancy was still possible in all cases, except for one sample where the tumor area was not situated within the scanned area (as shown in Table 1 under 3.5).

**Table 1:** Comparison between diagnostic outcome of FCM images and conventional histology

Case Number	Tissue	Tumor diagnosis Vivascope	Tumor diagnosis Histology	Resection margins Vivascope	Resection margins Histology
1	Surgical specimen	No tumor	Invasive carcinoma	Ill-defined, no tumor	No tumor
2	Surgical specimen	No tumor	No tumor	Ill-defined, no tumor	No tumor
3	Surgical specimen	No tumor	No tumor	Ill-defined, no tumor	No tumor
4	Surgical specimen	No tumor, adipose tissue	No tumor	Ill-defined, no tumor	No tumor
5	Surgical specimen	Invasive carcinoma	Invasive carcinoma	Ill-defined, no tumor	Ill-defined, no tumor
6	Surgical specimen	Invasive carcinoma (fragmented)	Invasive carcinoma (Out of focus)	Ill-defined, no tumor	Ill-defined, no tumor
7	Surgical specimen	Invasive carcinoma	Invasive carcinoma	Ill-defined, no tumor	DCIS
8	Surgical specimen	Invasive carcinoma	Invasive carcinoma	Ill-defined, no tumor	Ill-defined, no tumor
9	Surgical specimen	Invasive carcinoma	Invasive carcinoma	Ill-defined, no tumor	DCIS
10	Surgical specimen	Invasive carcinoma	Invasive carcinoma	Ill-defined, no tumor	Ill-defined, no tumor
11	Surgical specimen	Invasive carcinoma	Invasive carcinoma	Ill-defined, invasive Ca	No tumor
12	Surgical specimen	Invasive carcinoma	Invasive carcinoma	Ill-defined, no tumor	Ill-defined, no tumor
13	Surgical specimen	Invasive carcinoma	Invasive carcinoma	Ill-defined, no tumor	Ill-defined, no tumor
14	biopsy	No tumor	DCIS	n.a	n.a
15	biopsy	No tumor, intraductal proliferation	Ductal proliferation	n.a	n.a
16	biopsy	Invasive carcinoma	Invasive carcinoma	n.a	n.a
17	biopsy	Invasive carcinoma	Invasive carcinoma	n.a	n.a

### Staining properties

**Fig. 2:**
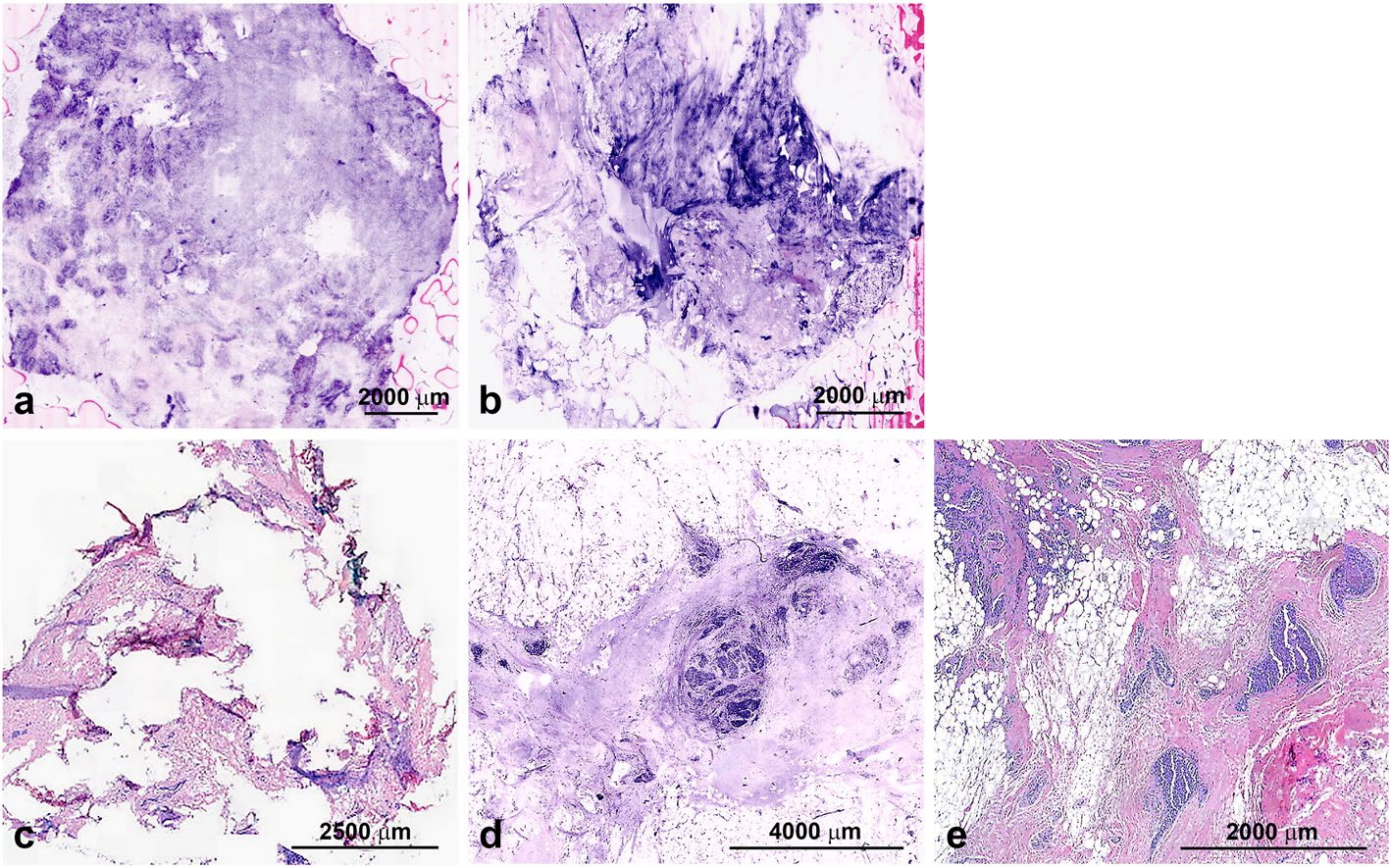
**A** Uneven staining, **B** Mechanical pressure too high in certain areas (middle) and too low in upper right hand corner, **C** Frozen Section of tumor area, **D** Vivascan 2500M-G4 image of tumor area from same case, **E** Traditional HE staining of tumor area from same case

**Fig. 3:**
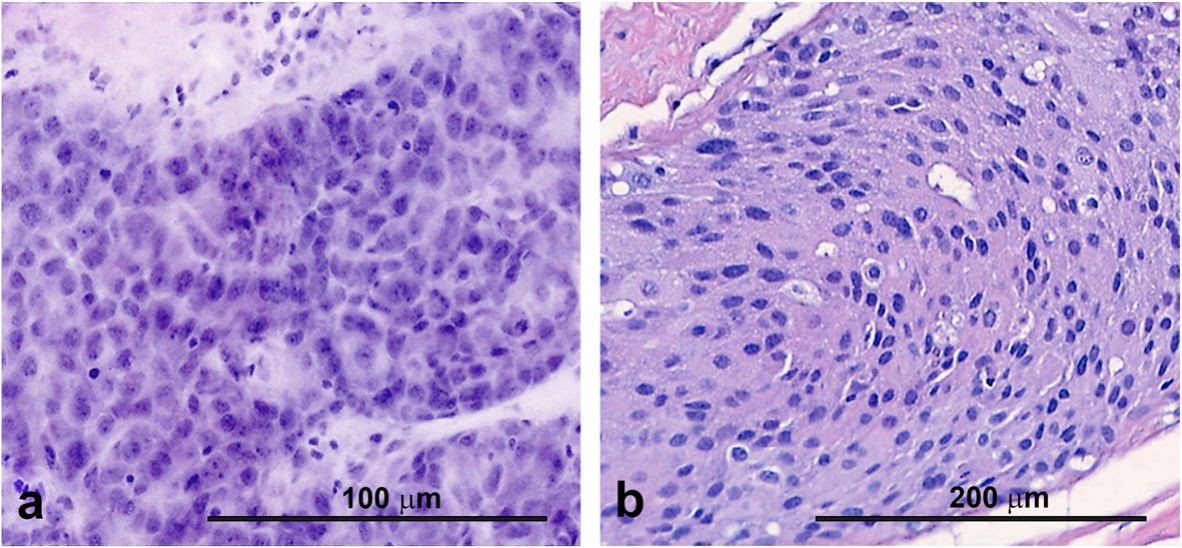
**A** FCM images of tumor cells in higher magnification, **B** Traditional H&E tumor cells in higher magnification

**Fig. 4:**
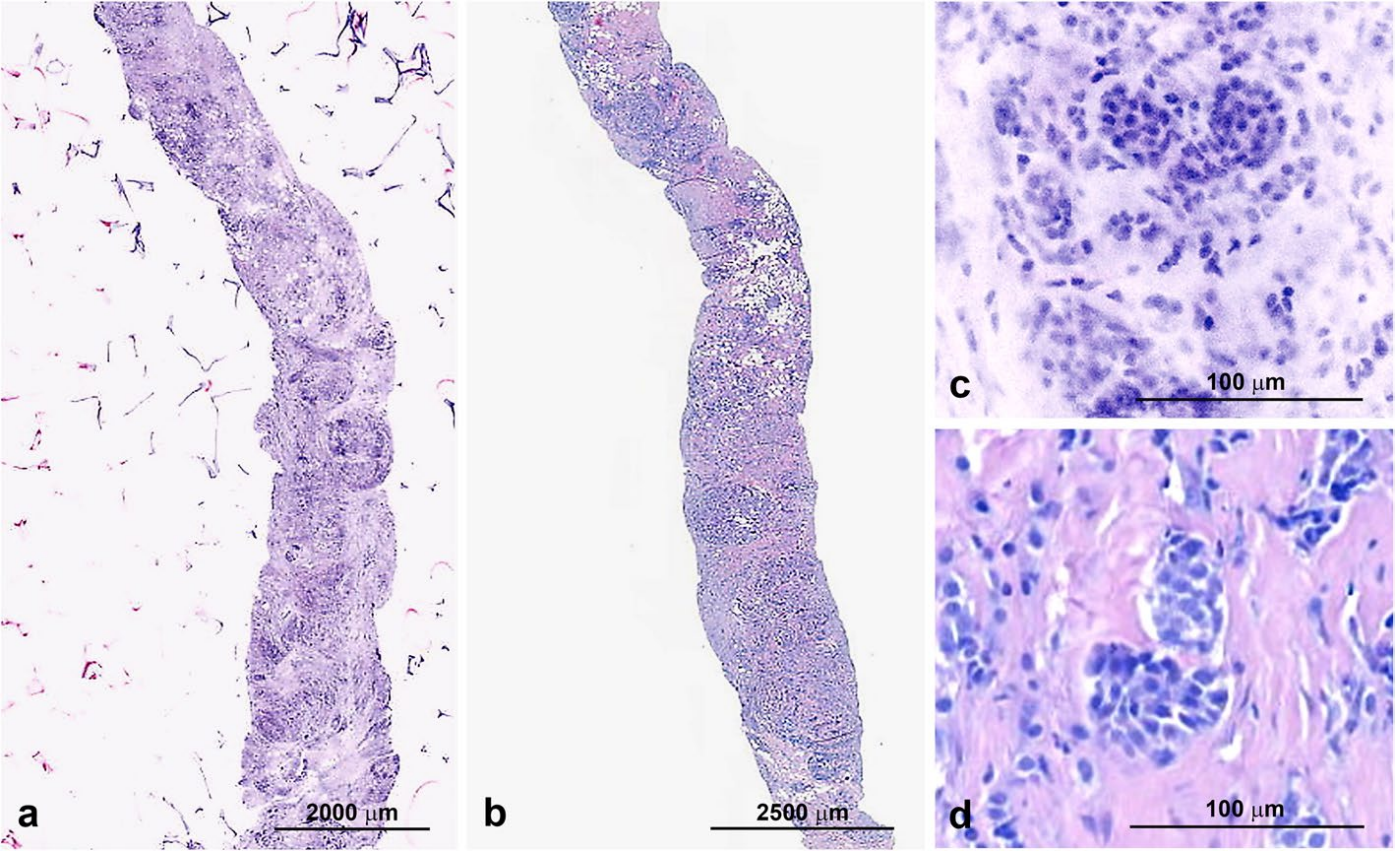
**A** Vivascope 2500 M-G4 biopsy, **B** traditional HE biopsy, **C** close-up of tumor cells in Vivascope 2500 M-G4, **D** and traditional HE

The staining properties on FCM images were comparable to that of standard HE staining. Distribution was patchier while colour and intensity were comparable to standard HE staining, being less intensely coloured in eosinophilic areas and had an overall deeper blue appearance in FCM (Figs. 2, 3, 4).

Margins of the tissue specimen were sometimes difficult to detect and ill-defined on FCM images due to lifting of the margins from the glass slide and increased blurriness in these areas. This was in part also due to the fact that ink applied to the surface of the specimens for identification of resection margins, cannot be visualized in FCM images.

### Morphologic detail

We obtained digital microscopic images from all 17 specimens investigated in this study. Due to the source of tissue (breast) the specimens consisted of connective tissue, glands and adipose tissue. Tissue architecture was generally better preserved in FCM virtual sections than in frozen sections especially in areas of adipose tissue. (Fig. 2). Nuclear morphology in FCM images is generally better than in frozen sections and almost matched the level of detail in FFPE sections. Visibility of cytoplasmic detail as well as cell borders was comparable to frozen sections but less well defined than in H&E stained FFPE sections.

While the integrity of adipose tissue is mostly lost in frozen sections, it is far better preserved in FCM images which is of particular interest since resection margins commonly are within adipose tissue. As seen in Fig. 2, the frozen section shows typical artefacts of tissues rich in adipose cells with fragmentation of the sample and no clearly defined tumor area. The FCM image analysis in comparison clearly shows areas of tumor cells which are also seen in FFPE section.

Figure 3 shows a higher magnification of tumor cells in the VivaScope® 2500 M-G4 analysis and the traditional HE staining. FCM yields a detailed depiction of the nuclear features comparable to the traditional HE.

### Comparison of final diagnoses

We compared diagnoses, defined as the presence or absence of tumor, using FCM with the standard H&E. In order to mimic clinical applications as closely as possible we additionally performed needle core biopsies from mastectomy specimens in four patients. Out of 17 analyzed tissue samples, 16 yielded the same positive tumor diagnosis (Table 1). Detailed analysis of the discrepant case revealed that the tissue was most likely flipped over for histologic analysis which means we looked at the opposite side of the specimen in the Vivascope 200 M-G4 analysis. Figure 3 shows an example of clearly distinguishable tumor cells in one of the samples.

## Discussion

To evaluate FCM in comparison to standard frozen sectioning, that often yields results of subpar quality in specimen rich in adipose tissue, with structural changes that might prevent correct diagnoses, we compared FCM with standard in-house procedures for frozen sections. This study was driven by two main intentions. One aspect was that we wanted to investigate the performance of FCM in specimens that are difficult to process in frozen sections. Another goal was assessing the suitability of FCM for rapid intraoperative telepathology which is of considerable interest for institutions without an in-house pathology lab. Many tumors are embedded in adipose tissue and a meaningful analysis regarding their size and exact location may not be possible in frozen sections in some cases since cryotome sectioning often leaves empty spaces in areas of adipose tissue.

Diagnostic accuracy and applicability of FCM has been shown to be excellent in a variety of studies: Bertoni et al. showed that diagnostic accuracy and learning curves were very good for pathologists in their study with prostatic tissue [7]. Schüürmann et al. also showed a high correlation between the digital images and the H&E-stains in their study on skin samples [12]. Our study confirmed the very good diagnostic accuracy for detecting malignancy (16 out of 17 cases yielded the same result compared to H&E analysis. The only discordant case was scanned in different orientation and therefore did not depict the relevant tumor area. Due to the H&E-like pseudo-staining of the images, interpretation was easy and intuitive for the pathologists.

A very promising perspective of FCM is intraoperative telepathology, which is gaining increasing relevance as pathology departments around the world are moving towards fewer, more specialized diagnostic centers [13] and considering the shortage of pathologists in rural areas that is likely to increase in the future: Tissues can be processed in one institution that does not require an on-site pathology department or equipment and can then be analyzed in a remote specialized center that theoretically can be located anywhere in the world.

A relevant aspect that needs to be assessed when considering FCM as an alternative to frozen sections is the duration of the procedure. The staining and scanning procedure with the VivaScope® 2500 M-G4 was 4–6 min and thus comparable to an average frozen section. The whole procedure is entirely carried out by one person.

The tissue samples submitted for frozen section analysis are typically first assessed by the pathologist and then transferred to a lab technician for freezing, sectioning and staining. The tissue processing is carried out by a skilled pathologist/technician-team typically and requires at least 4–6 min. In case of adipose tissue or other specimens that are difficult to process in frozen section histology this timeframe might be far exceeded.

While scanning time can vary based on sample size and settings, the entire procedure was usually at least comparable and in cases with very small samples slightly faster than the average duration of frozen section.

As shown above the quality of the scanned tissue samples is comparable to the frozen sections but is inferior to that of FFPE sections especially in terms of cytoplasmic details and structure of extracellular matrix.

The loss of tissue for subsequent analysis due to frozen sections can put the pathologist in a significant predicament in cases when diagnosis from paraffin-embedded material without frozen section artefacts is required and the frozen section is needed for intraoperative guidance of resection. An important benefit of FCM is that no tissue is lost due to sectioning and the entire specimen remains available for subsequent analyses which is of particular importance for small samples. In addition tissue does not get frozen and can therefore be treated like a normal specimen in subsequent analyses without the risk of freezing artefacts. Mechanical alterations of the tissue introduced by the scanning process were not detectable and AO staining did not affect subsequent processing for conventional FFPE histology. We however did not assess a potential impact of tissue processing for FCM on molecular pathology analyses.

We found that FCM is an efficient and promising alternative to frozen sections especially if specimens containing adipose tissue. It adequately portrays areas of interest (tumor areas, adipose tissue) and correct assessment of presence or absence of tumor was possible in 16 out of 17 cases with one case being scanned with insufficient quality to perform an adequate diagnosis.

One of the drawbacks of FCM is that depiction of extracellular matrix is somewhat different to the pattern seen in H&E histology, which might, in certain tissues, obscure the interaction of tumors with the surrounding stroma. In our study, we however did not experience incorrect assessment of tumor invasion or the lack thereof. Further research into adequate depiction of extracellular matrix should be conducted in future studies in order to achieve an even closer match of FCM images with H&E histology. Another limitation of FCM that was more relevant for our study is the representation of resection margins: We sometimes observed ill-defined borders of the specimens most likely due to lifting of margins. The inability to visualize ink applied to the specimen’s surface for marking resection margins also complicated the exact measurement of the distance of tumor cells from the resection margin. This might be a serious issue in cases where the tumor is very close to the margin.

In conclusion, we found that when comparing frozen sections to FCM images generated with the VivaScope® 2500 M-G4, FCM images had greater tissue integrity, morphological detail and an adequate quality of staining not only in regards to adipose tissue but also tumor areas with a weakness in depicting the sample margins. Despite this drawback, the VivaScope® 2500 M-G4 has the potential to expand the application of rapid tissue analysis as speed and quality potentially exceeds that of frozen sections especially in tissues rich in adipose cells as is the case of breast tissue.

## Data Availability

The datasets used and/or analysed during the current study are available from the corresponding author on reasonable request. The datasets supporting the conclusions of this article are included within the article (and its additional files).
